# Loureirin B inhibits the proliferation of hepatic stellate cells and the Wnt/β-catenin signaling pathway by regulating miR-148-3p

**DOI:** 10.1186/s11658-018-0098-9

**Published:** 2018-08-02

**Authors:** Jian-Peng Hu, Rong Zhang, Min Tang, Yu-Lian Li, Lin-Ting Xun, Zhi-Zhou Shi, Ying An, Ting Li, Zheng-Ji Song

**Affiliations:** 1 Medical School, Kunming University of Science and Technology, Kunming, 650500 Yunnan province China; 2 Department of Gastroenterology, the First People’s Hospital of Yunnan province, the Affiliated Hospital of Kunming University of Science and Technology, Kunming, 650032 Yunnan province China

**Keywords:** Loureirin B, Liver fibrosis, Wnt1, miR-148-3p, Hepatic stellate cells

## Abstract

**Background:**

We investigated the activity of loureirin B against liver fibrosis and the underlying molecular mechanisms.

**Methods:**

Hepatic stellate cells (HSCs) from Sprague-Dawley rats were treated with different concentrations of loureirin B. We used the MTT assay to determine HSC proliferation, flow cytometry to analyze apoptosis, and western blot to determine the expressions of Bax, Bcl-2, Wnt1 and β-catenin. Real-time PCR was used to determine the expressions of Wnt1 and miR-148-3p.

**Results:**

The MTT assay showed that loureirin B treatment significantly inhibited the proliferation of HSCs in time- and dose-dependent manners. Loureirin B significantly promoted the apoptosis of HSCs, increased the expression of Bax and decreased the Bcl-2 level. Western blot analysis showed that the expressions of Wnt1 and β-catenin were obviously lower in the loureirin B treatment group than in the control group. We also found that loureirin B could decrease the Wnt1 mRNA level and increase miR-148-3p expression. Knockdown of miR-148-3p using inhibitor could reverse the effects of loureirin B on the proliferation and apoptosis of HSCs and the expressions of Bax, Bcl-2, Wnt1 and β-catenin.

**Conclusion:**

Our results suggest that loureirin B inhibited the proliferation and promoted the apoptosis of HSCs, and suppressed the Wnt/β-catenin signaling pathway via regulation of miR-148-3p.

## Background

Liver fibrosis is caused by repeated healing, repair and interstitial reconstruction after chronic liver injury. The activation of hepatic stellate cells (HSCs) is a critical event in liver fibrosis because these cells are the primary source of extracellular matrix in the injured liver [1]. Promoting HSC apoptosis and inhibiting HSC activation, proliferation and extracellular matrix production are important therapeutic approaches in cases of liver fibrosis. As the Wnt/β-catenin signaling pathway plays a vital role in the proliferation, differentiation, division, migration and apoptosis of HSCs, aberrant activation of this pathway may induce hepatic fibrogenesis. It was recently reported that blocking the Wnt/β-catenin signaling pathway is a crucial method to treat hepatic fibrogenesis [2, 3].

Wnt1 is a key member of the canonical Wnt/β-catenin signaling pathway, which activates the disheveled proteins, inhibits GSK-3 kinase, and accumulates β-catenin in the nucleus, and finally activates the β-catenin target genes. In this study, we explored the roles and molecular mechanisms for loureirin B in liver fibrosis. Our results indicate that loureirin B inhibited the proliferation and promoted the apoptosis of activated HSCs, and inhibited the Wnt signaling pathway via regulation of miR-148-3p.

## Methods

### Preparation, culture, identification and treatment of HSCs

Sixteen 400- to 450-g male Sprague-Dawley rats (specific pathogen free, from the Laboratory Animal Center of Chinese Academy of Sciences) were used in this study. Isolation of HSCs was performed as previously described [4]. Briefly, the liver was digested and perfused with collagenase IV (Worthington Biochemical Corporation), chain protease E and DNaseI solution, and then HSCs were isolated from the non-parenchymal liver cell (NPLC) fraction using 12% Nycodenz density gradient centrifugation (Nycodenz).

The primary HSCs were cultured in Dulbecco’s modified Eagle’s medium (DMEM) High Glucose (Gibco) supplemented with 20% fetal bovine serum (FBS; GE Healthcare Life Sciences), 100 μg/ml penicillin and 100 μg/ml streptomycin, at 37 °C in a humid incubator containing 5% CO_2_. HSCs were identified via vitamin A lipid droplet auto-fluorescence using a fluorescence microscopy (Nikon Corporation), and HSC viability and purity were found to be above 90%. Activated HSCs were identified with immunofluorescence staining of α-SMA, which was the marker of activated HSCs after 1 week [5], and then treated with loureirin B. This study was approved by the Medical Ethics Committee of the First People’s Hospital of Yunnan Province.

### Loureirin B and inhibitor of miR-148-3p

Loureirin B was obtained from the National Institute for the Control of Pharmaceutical and Biological Products of China, and reconstituted in DMSO at a final stock concentration of 20 mg/ml. The inhibitor of miR-148-3p was produced by GenePharma.

### Detection of cell proliferation

Activated HSCs were cultured at a density of 10^5^ per ml in DMEM High Glucose supplemented with 10% FBS in 96-well plates (100 μl/well), and then treated with 12.5, 25, 50, 100, 200 or 400 ng/μl loureirin B (concentrations based on the results of previous studies) [6, 7]. Two hours before the end of the incubation, 20 μl of the above-mentioned MTT solution was added to each well. Incubation was at 37 °C for 2 h. The inhibitory rates of activated HSC (aHSC) proliferation were determined by measuring absorbance at 570 nm.

### Detection of apoptosis

The Apoptosis Detection Kit (BD Biosciences) was used to detect the apoptosis of HSCs. Activated HSCs that were treated with loureirin B were washed twice with cold PBS, and then suspended using 1× binding buffer at a concentration of 1 × 10^6^ cells/ml. Then 100 μl of the solution (1 × 10^5^ cells) was added to 5 ml culture, followed by 5 μl PI and 5 μl of FITC Annexin V. This was incubated for 15 min at room temperature in the dark, then 400 μl of 1× binding buffer was added to each tube. Analysis was done with a FACScan Cytometer (BD Accuri C6; BD Biosciences).

### Western blot analysis

Specific antibodies of Wnt1, β-catenin, Bax and Bcl-2 (Santa Cruz Biotechnology) were used for western blot. HSCs were washed three times with ice-cold PBS and lysed in 100 to 500 μl RIPA lysis buffer (p0013B, Beyotime, supplemented with 1% PMSF). Cell debris was removed by centrifugation at 13,000×g for 30 min at 4 °C. Protein samples were boiled with 5× SDS-PAGE loading buffer for 10 min and subjected to electrophoresis in denaturing 10% SDS-polyacrylamide gel. Then proteins were transferred onto a polyvinylidene fluoride membrane (Millipore). The membrane was washed with Tris-buffered saline Tween (TBST), and blocked with 5% bovine serum albumin (BSA). The image was quantitatively analyzed using FluorChem V2.0 software.

### Real-time PCR

Real-time PCR was performed to detect the relative expression level of Wnt1. PCR was performed in a total volume of 20 μl, including 10 μl of 2× Power SYBR Green PCR Master Mix (Applied Biosystems), 2 μl of cDNA (5 ng/μl) and 1 μl of primer mix (10 μM each). PCR amplification and detection were performed in a LightCycler 480 II (Roche Applied Science) as follows: an initial denaturation at 95 °C for 10 min; and 40 cycles of 95 °C for 15 s and 60 °C for 1 min. The relative gene expression was calculated using the comparative C_T_ Method. The gene expressions of the target gene were normalized to an endogenous reference (GAPDH), and their values relative to the calibrator were obtained with the formula 2^−ΔΔCt^. ΔC_T_ was calculated by subtracting the average GAPDH C_T_ from the average C_T_ of the gene of interest. The ratio defines the level of relative expression of the target gene to that of GAPDH.

Hairpin-it miR-148-3p qRT-PCR Primer Set (GenePharma) was used for the measurement of the relative quantity of miR-148-3p. The mRNA expression of miR-148-3p was normalized to the endogenous expression of U6.

### Statistical analysis

Statistical analyses were performed using SSPS18.0 software. Data are presented as means ± standard deviations (ranges). The differences between the control group and treatment group were compared with the Student’s t-test. *p* < 0.05 was regarded as statistically significant.

## Results

### Separation and identification of HSCs

We used an inverted phase contrast microscope to observe the primary quiescent HSCs separated from the rat liver. It is easy to see the spontaneous green fluorescence, but it disappeared quickly. The cells also contained many refractive granules. After culture for about 1 week, the HSCs changed their phenotype from quiescent to activated. Immunofluorescence staining showed that the positive expression of α-smooth muscle actin (α-SMA) was above 90%, and the shapes of aHSC changed to a star (not shown).

### Loureirin B inhibits the proliferation and promotes the apoptosis of HSCs and downregulates the expression of Bcl-2

**Fig. 1:**
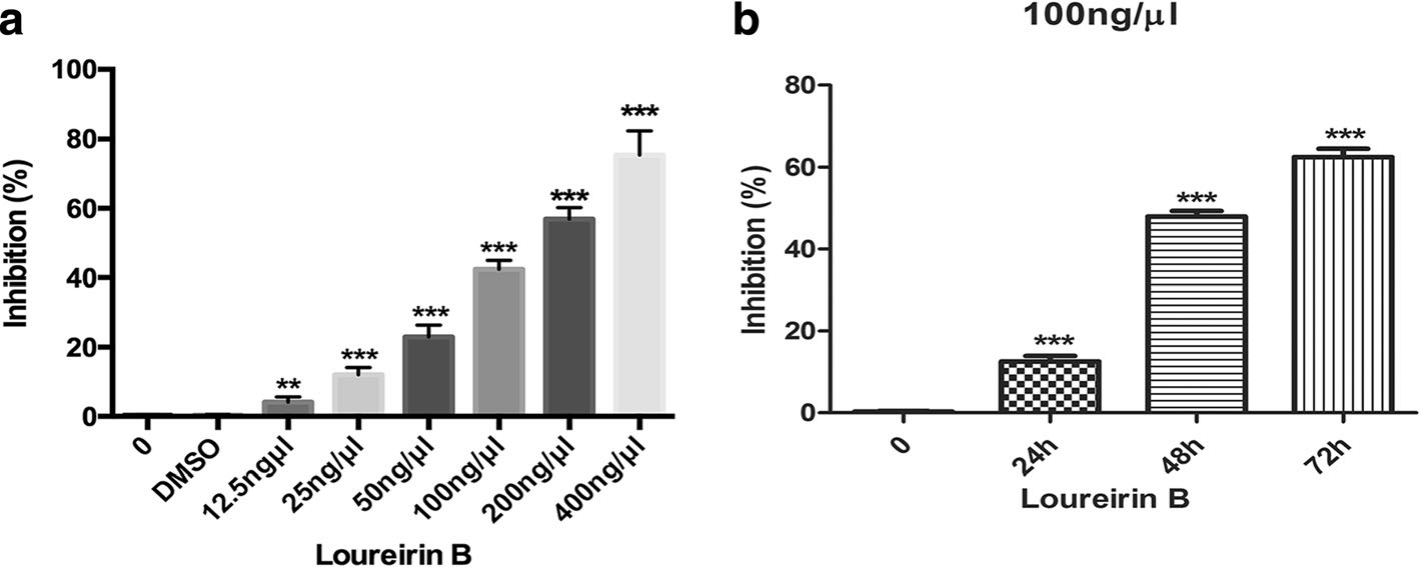
Loureirin B inhibits the proliferation of HSCs. **a** Activated hepatic stellate cells (aHSCs) were treated with 12.5, 25, 50, 100, 200 or 400 ng/μl loureirin B for 48 h, and then detected using the MTT assay. **b** aHSCs were treated with 100 ng/μl loureirin B for 24, 48 and 72 h, and then detected using the MTT assay. The experiments were repeated three times. *p < 0.05; **p < 0.01; ***p < 0.001

**Fig. 2:**
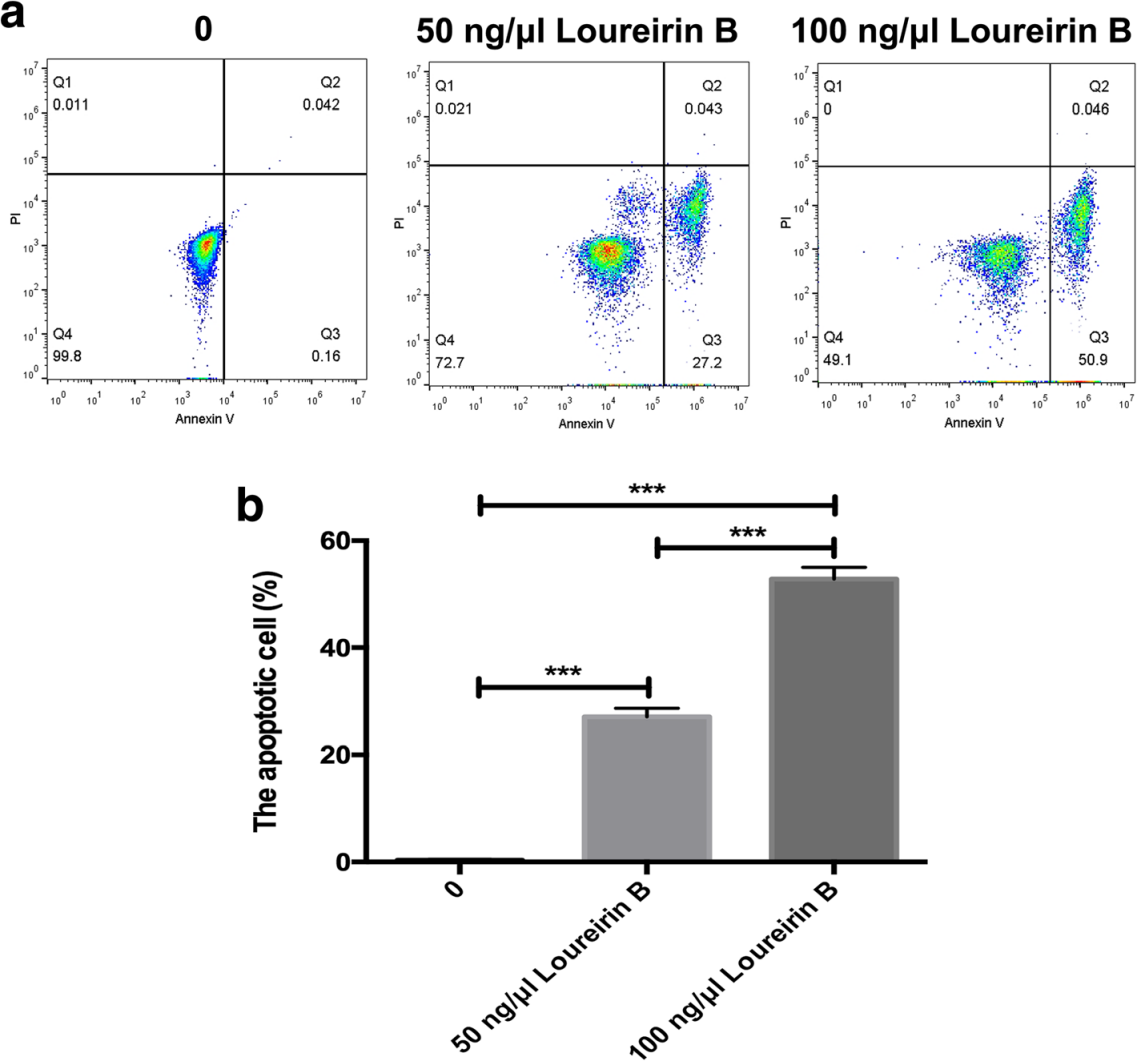
Loureirin B promotes the apoptosis of HSCs. **a** Activated hepatic stellate cells (aHSCs) were treated with 50 and 100 ng/μl loureirin B for 48 h, and then assessed using flow cytometry. **b** Statistical analysis of flow cytometry results. Data are the means ± SEM from four independent experiments. *p < 0.05; **p < 0.01; ***p < 0.001

**Fig. 3:**
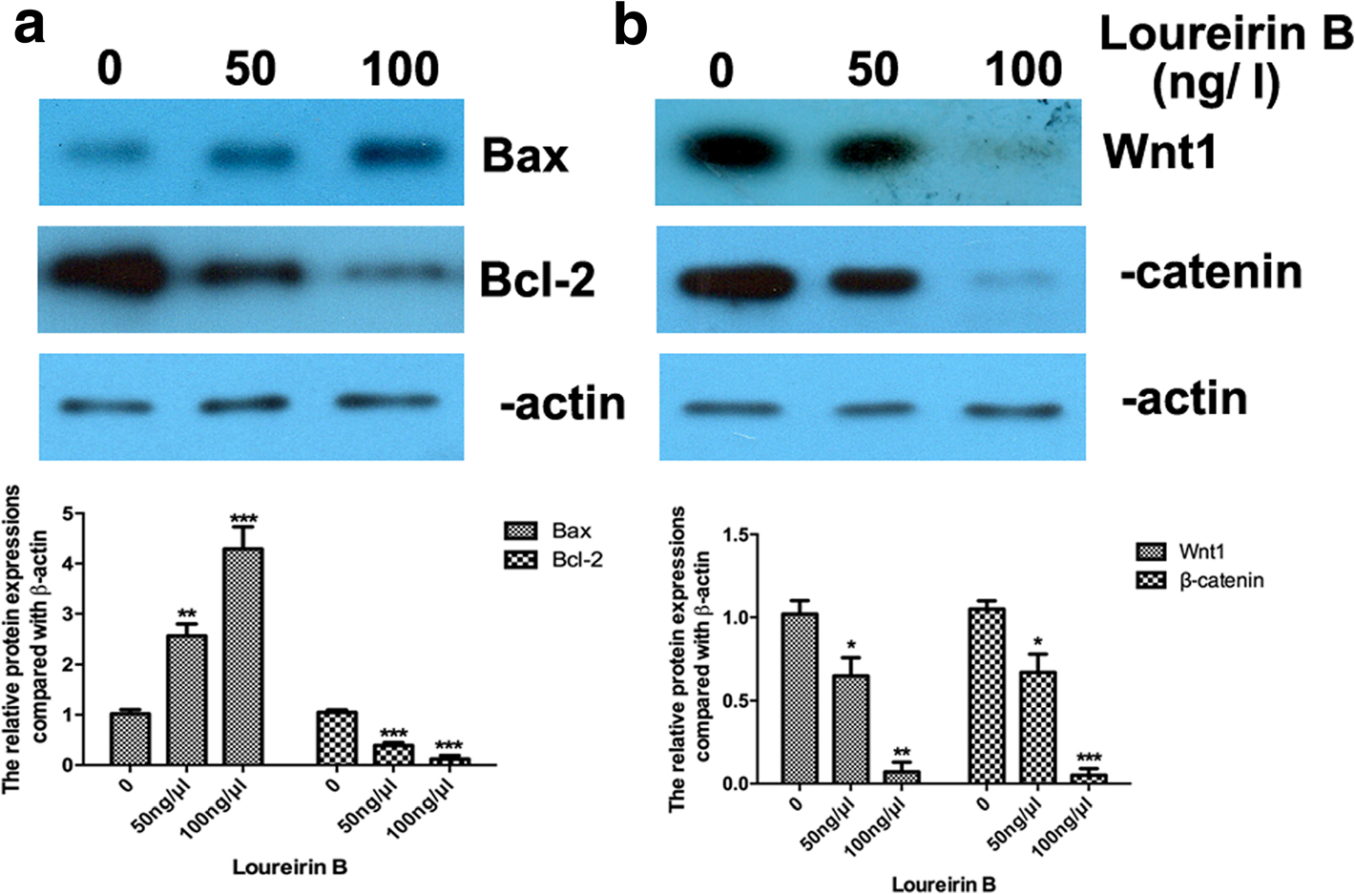
Loureirin B increased Bax expression, decreased Bcl-2 expression, and inactivated the Wnt/β-catenin signaling pathway. **a** and **b** Effect of loureirin B on the expression levels of Bax, Bcl-2, Wnt1 and β-catenin, as detected via western blot assay and statistically analyzed using Student’s t-test. Data are the means ± SEM from four independent experiments. *p < 0.05; **p < 0.01; ***p < 0.001

We treated the HSCs with loureirin B, and found that it significantly inhibited their proliferation in a dose-dependent manner (Fig. 1a). We also assessed the inhibitory effects of loureirin B in different time, and the results showed that loureirin B also inhibited the proliferation of HSCs in a time dependent manner (Fig. 1b). Using flow cytometry assay, we further found that loureirin B could promote the apoptosis of HSCs in a dose dependent manner (Fig. 2a and b). Western blotting assay showed that loureirin B increased the expression of Bax and decreased the level of Bcl-2, and the expression of Bcl-2 in 100 ng/μl loureirin B group was lower than that in 50 ng/μl loureirin B group (Fig. 3a).

### Loureirin B inhibits the proliferation and promotes the apoptosis of HSCs, and inhibits the Wnt/β-catenin signaling pathway via regulation by regulating miR-148-3p

**Fig. 4:**
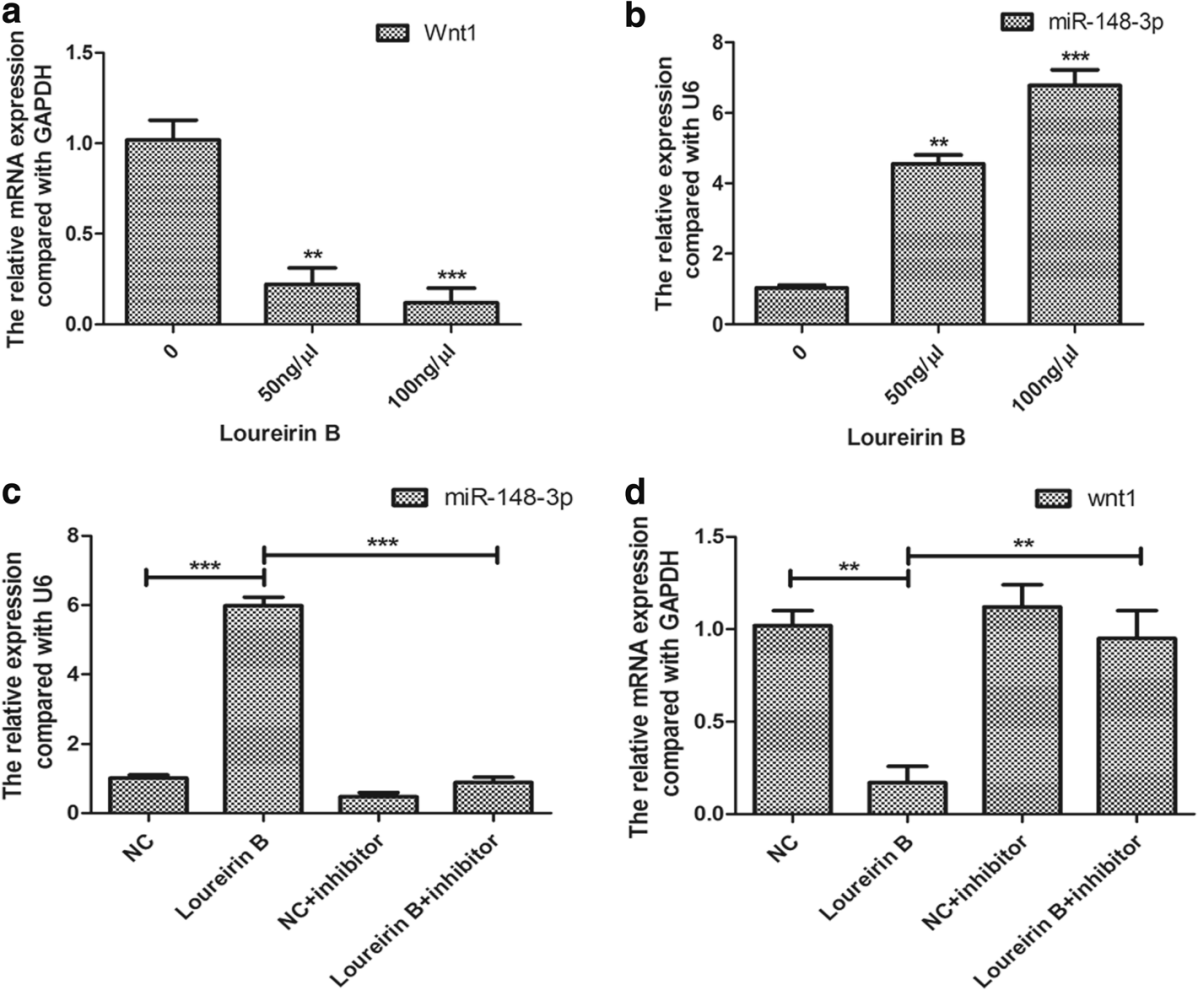
Loureirin B decreased the expression of Wnt1 via upregulation of miR-148-3p. **a** and **b** The expression levels of Wnt1 and miR-148-3p were detected using real-time PCR. **c** Using the miR-148-3p inhibitor to silence miR-148-3p. **d** Knockdown of miR-148-3p increased the loureirin B-decreased Wnt1 mRNA level detected using real-time PCR. Data are the means ± SEM from four independent experiments. *p < 0.05; **p < 0.01; ***p < 0.001

Activating the Wnt/β-catenin signaling pathway was reported to promote liver fibrosis [8], so we explored whether loureirin B affected this pathway. Our western blot results showed that loureirin B significantly reduced the protein expression levels of Wnt1 and β-catenin (Fig. 3b), and that 100 ng/μl loureirin B had a higher inhibitory effect on the expressions of Wnt1 and β-catenin than 50 ng/μl loureirin B (Fig. 3b). Using real-time PCR, we found that loureirin B also decreased the mRNA level of Wnt1 (Fig. 4a). Using TargetScan, we predicted that miR-148-3p could target Wnt1.

**Fig. 5:**
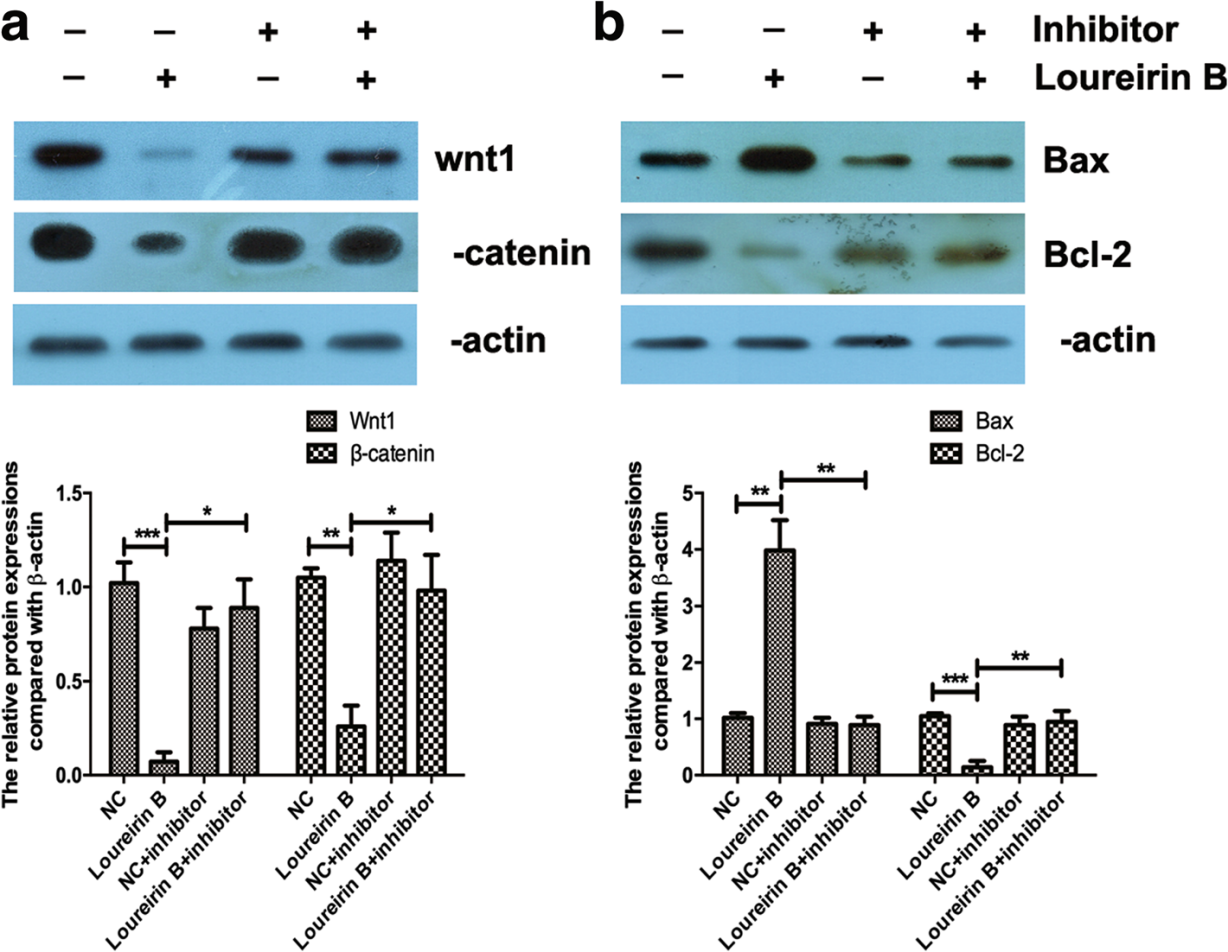
Loureirin B regulated the Wnt/β-catenin signaling pathway and the expressions of Bax and Bcl-2 via upregulation of miR-148-3p. **a** Knockdown of miR-148-3p increased the loureirin B-decreased Wnt1 and β-catenin, as detected via western blot. **b** Knockdown of miR-148-3p downregulated the loureirin B-increased Bax and upregulated the loureirin B-decreased Bcl-2 expression. Data are the means ± SEM from four independent experiments. *p < 0.05; **p < 0.01; ***p < 0.001

Then we detected the expression of miR-148-3p in the HSCs treated with loureirin B. The results showed that loureirin B significantly increased miR-148-3p expression (Fig. 4b). To confirm that loureirin B inhibited the Wnt/β-catenin signaling pathway via regulation of miR-148-3p, we used an inhibitor to reduce its expression in the loureirin B-treated HSCs (Fig. 4c). The results showed that knockdown of miR-148-3p increased the Wnt1 mRNA and protein levels that had been reduced by loureirin B treatment (Figs 4d and 5a). We also found that silencing the miR-148-3p in loureirin B-treated cells could reverse the effects of loureirin B on the expressions of β-catenin, Bax and Bcl-2 (Fig. 5a and b).

**Fig. 6:**
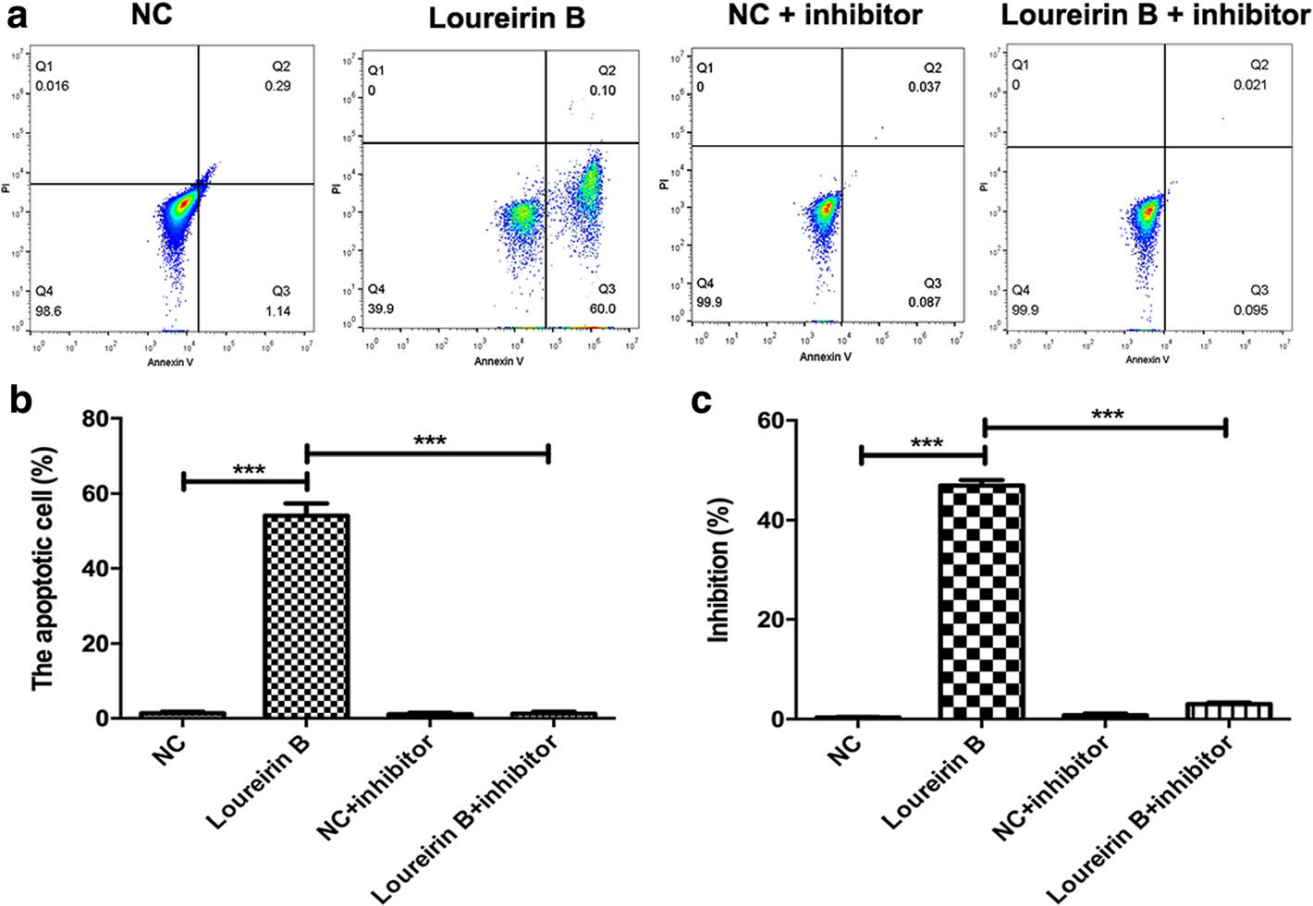
Loureirin B inhibits the proliferation and promotes the apoptosis of HSCs via regulation of miR-148-3p. **a** and **b** Flow cytometry was used to analyze the apoptosis of HSCs when inhibitor was applied to silence the miR-148-3p expression. **c** The proliferation of HSCs was detected using the MTT assay. Data are the means ± SEM from four independent experiments. *p < 0.05; **p < 0.01; ***p < 0.001

These results indicate that loureirin B inhibited the Wnt/β-catenin signaling pathway via regulation of miR-148-3p. Silencing miR-148-3p could reduce loureirin B-induced apoptosis and enhance loureirin B-suppressed proliferation of HSCs (Fig. 6a and b). These results indicate that loureirin B affected the proliferation and apoptosis of HSCs via regulation of miR-148-3p.

## Discussion

The activation of HSCs is considered an important event in liver fibrosis, and epithelial–mesenchymal transition (EMT) plays an important role in HSC activation [9]. HSCs have several important functions under both physiological and pathological conditions associated with sinusoids [10]. In the healthy liver, HSCs have two morphologically distinguishable forms: the quiescent HSCs characterized by small cell size and low proliferating activity [11]; and the activated form [12, 13]. Under physiological conditions, quiescent HSCs store about 75% of total body vitamin A in lipid droplets in the cytoplasm, participate in the metabolism of extracellular matrix in liver sinusoids, and regulate the bleeding functions of sinusoids [10, 14, 15]. In response to liver injury, HSCs change from quiescent to activated [12, 13], with this transition is caused by the inflammatory factors, such as TGF-β1, PDGF, VEGF, TNF and so on, which were produced by immune cells, Kupffer cells and endothelial cells. Activated HSCs proliferate vigorously, lose vitamin A, express α-SMA, and synthesize a large amount of extra-cellular matrix (ECM) components [16]. The activated HSCs also change to fibroblasts or myofibroblasts [17]. It is clear that TGF-β1 could promote HSC activation [18]. HSCs could produce and secrete TGF-β1 through autocrine and paracrine manners, while TGF-β1 could promote HSC proliferation and accumulation of ECM in the liver, leading to liver fibrosis [19].

Resina draconis is a special resin derived from the fruit and stem of certain palm trees, such as *Dracaena cochinchinensis* (Lour.) S. C. Chen, with the efficacy of promoting blood circulation and angiogenesis, diminishing inflammation, relieving pain and stopping bleeding. Loureirin B is one of the most important chemical compositions and physiologically active ingredients of resina draconis. It has the molecular structure propan-1-one, 1-(4-hydroxyphenyl)-3-(2, 4, 6-trimethoxyphenyl)- 1-(4-hydroxyphenyl)-3-(2, 4, 6-trimethoxyphenyl) propan-1-one.

Jiang et al. reported that loureirin B was the inhibitor of PAI-1 (IC_50_ = 26.10 μM) which was the natural inhibitor of tissue-type and urokinase-type plasminogen activators. Loureirin B could inhibit the formation of the PAI-1/uPA complex [20]. Loureirin B could promote insulin secretion by upregulating the mRNA expressions of Pdx-1, MafA and the intracellular ATP level, and inhibiting the K_ATP_ current [17]. He et al. reported that loureirin B downregulated p-ERK and p-JNK in TGF-β1-stimulated fibroblasts and cultured hypertrophic scar tissue ex vivo, and further affected the expressions of Col1 and FN [6]. In hypertrophic scar fibroblasts, loureirin B could dose-dependently reduce the mRNA and protein levels of type I collagen (CoII), type III collagen (ColIII) and α-smooth muscle actin (α-SMA) by regulating MMPs and TIMPs, inhibit scar fibroblast proliferation and suppress TGF-β1-induced fibrosis, possibly via TGF-β1/Smad2/3 pathway [7]. Our study found that loureirin B inhibited HSC proliferation, promoted Hsc apoptosis, and suppressed the Wnt signaling pathway via regulating miR-148-3p.

The Wnt/β-catenin signaling pathway plays important roles in HSC activation [21]. Wnt1 is an essential component of the canonical Wnt/β-catenin signaling pathway, which leads to the activation of disheveled proteins, inhibition of GSK-3β kinase, nuclear accumulation of β-catenin, and finally activation of Wnt target genes [22]. Inhibiting the expression of Wnt1 maybe an attractive method to block the Wnt/β-catenin signaling pathway, thereby inhibiting the activation of HSCs.

## Conclusions

Our findings indicate that loureirin B inhibited HSC proliferation, promoted HSC apoptosis, and downregulated the expression of Bcl-2. Very interestingly, loureirin B inhibited the Wnt/β-catenin signaling pathway via regulation of miR-148-3p in HSCs.

There have been no reports about the correlation between miR-148-3p and HSC function. Our results found that miR-148-3p was involved in the regulation of lourerin B on HSC proliferation and apoptosis. Our findings suggest that three major aspects of loureirin B activity – inhibition of HSC proliferation, promotion of HSC apoptosis, and inactivation of the Wnt/β-catenin signaling pathway – occur via regulation of miR-148-3p.

## Abbreviations

ECM: Extra-cellular matrix
HSCs: Hepatic stellate cells
NPLCs: Non-parenchymal liver cells
α-SMA: α-smooth muscle actin
